# From Disciplinary Societies to Societies of Control: Foucault, Deleuze and the Care of People Under the Mental Health Review Board in Psychiatric Settings

**DOI:** 10.1111/inm.70220

**Published:** 2026-01-20

**Authors:** Etienne Paradis‐Gagné, Marie‐Ève Roy‐Ducharme, Manouel Argod, Emmanuelle Bernheim, Dave Holmes

**Affiliations:** ^1^ Faculty of Nursing Université de Montréal Montreal Québec Canada; ^2^ Canada Research Chair on Mental Health and Access to Justice, Faculty of Law, Civil Law Section University of Ottawa Ottawa Ontario Canada; ^3^ School of Nursing, University of Ottawa Ottawa Ontario Canada

**Keywords:** care partnership, grounded theory, judiciarization, mental health nursing, not criminally responsible, societies of control

## Abstract

The number of individuals declared not criminally responsible on account of mental disorder is increasing in Canada. The supervision of these individuals—balancing the imperatives of public safety with philosophies of care—can create significant ethical dilemmas for psychiatric nurses. This article examines the intersection of mental health and the justice system, drawing on the accounts of nurses and service users admitted to psychiatric units. Using grounded theory, the study seeks to deepen understanding of the impact of judiciarization, particularly its influence on therapeutic relationships and the perceived quality of care. Special attention is given to the role of the Mental Health Review Board (MHRB) in monitoring individuals found NCRMD. Grounded in the theoretical framework of Deleuze's notion of societies of control, the analysis of interview data reveals three central categories: (1) the process of involuntary (re)admission; (2) the complexities of the care partnership; and (3) ethical tensions and prejudice. The experiences of users are explored through the lenses of coercive practices, disciplinary structures, and varied forms of social control in the context of MHRB supervision. Psychiatric nurses' narratives point to challenges related to power imbalances, conflicts of professional allegiance, and ambivalence in care perspectives—highlighting the need for ongoing ethical reflection and systemic change in the management of NCR patients.

## 1 Introduction

In recent years, the number of individuals found not criminally responsible (NCR) on account of mental disorder has increased across Canada (Department of Justice 2015; Latimer and Lawrence 2006). This trend is particularly pronounced in Quebec (Crocker et al. 2015), where individuals found NCR are overseen by the Mental Health Review Board (MHRB) (Tribunal administratif du Québec (TAQ) 2024a). Under a review board order, NCR individuals may be detained in custody in a psychiatric hospital—either with or without conditions governing their potential release—or may be granted a conditional or unconditional discharge into the community, based on the level of risk they pose. Furthermore, hospitals may be granted delegated authority to readmit individuals on conditional release should their risk to public safety increase (Bernheim et al. 2023; TAQ 2024b).

Internationally, research has been conducted on care in the context of supervision by review boards. The literature indicates that rates of psychiatric readmission for NCR individuals are higher in young men (Salem et al. 2016), with repeated previous psychiatric admissions (Fazel et al. 2016), severe mental disorders linked to a history of substance abuse, and a weak support network (Almeida et al. 2016; Jewell et al. 2018). In terms of gender, it is reported that men are overrepresented with regard to NCR verdicts (Latimer and Lawrence 2006). Manguno‐Mire et al. (2014) note that the main reasons for readmission are substance use, non‐compliance with pharmacological treatment, failure to attend outpatient appointments, and violation of release conditions—similar findings have also been reported in Canada (Bernheim et al. 2023; Martin et al. 2022). Australian authors Lyons et al. (2022) also point to the presence of external stressors in the community (e.g., job insecurity or loss, lack of social support), and the progression of mental health symptoms as contributing factors to involuntary readmission. In their study, more than half of outpatients (*n* = 140, 57%) breached their conditions and had to be readmitted to a psychiatric institution.

Review boards monitor individuals primarily from a safety and public protection perspective (Latimer and Lawrence 2006; Sallée et al. 2022), which may conflict with the philosophies of care and recovery advocated in clinical settings. This can generate ethical and moral tensions among care providers who work with justice‐involved individuals and who find themselves in a dual role: caring and, at the same time, controlling. Navigating these opposing responsibilities can negatively impact the therapeutic relationship (Rye et al. 2019) and confront care providers with significant ethical dilemmas (Austin et al. 2009). As a result, relationships between nurses and justice‐involved patients are inherently complex (Gustafsson et al. 2013), shaped by coercion and power dynamics, particularly in psycho‐legal contexts (Marshall and Adams 2018).

Considering the various issues raised in the literature, we conducted a qualitative study on the interaction between mental health and justice systems, focusing on psychiatric nurses and patients admitted to a psychiatric institution. The aim of this project was to better understand how the judicial process affects people with mental disorders, as well as the nurses who work with them. We also sought to understand how this process influences the therapeutic relationship and the quality of care received by a population with complex health needs. The results of the larger study have been published (Paradis‐Gagné et al. 2023, 2024). For the purpose of this article, we analyse more specifically the data relating to persons under MHRB supervision, in order to better understand how this medico‐legal trajectory influences their daily realities.

## 2 Methods

### 2.1 Design

For this study, we used the qualitative grounded theory [GT] approach proposed by Corbin and Strauss (2014). This is a systematic method that differs in some respects from the classic approach of Glaser and Strauss (1967), being more guided and procedural. It is an inductive research design (Corbin and Strauss 2014), meaning that data collection and analysis are carried out simultaneously, in an iterative way. This approach focuses on social phenomena and processes, and on the social interactions between the actors who experience the phenomenon under study. This project was conducted in a psychiatric hospital in the province of Quebec, Canada.

### 2.2 Recruitment and Sampling

The theoretical sampling method (Draucker et al. 2007) was used for the project. In the GT approach, participants from diverse groups are selected to validate the categories and concepts emerging from the empirical data. The research was presented and an information document circulated to nurses and managers at team meetings in the units. Interested participants then contacted the research team to schedule an interview. For the recruitment of users, discussions were held on the units with nursing staff to identify potential participants whose clinical condition was stable enough to take part in the research. We stopped recruitment after reaching theoretical saturation (Corbin and Strauss 2014), that is, when the central categories were fully developed and it was no longer necessary to collect more data. Data collection took place between August 2022 and June 2023 and was carried out through semi‐structured interviews with admitted patients and nurses who practice in this setting. Interviews, lasting between 20 and 60 min, were conducted by the first author on the units and notes were taken. From the total sample (*n* = 19), we specifically analysed data relating to MHRB follow‐up, or what participants referred to as ‘TAQ’ [Tribunal Administratif du Québec]. We selected interviews with users (Table 1) admitted specifically under a MHRB mandate (*n* = 4) and nurses (Table 2) with experience in this context (*n* = 8).

**TABLE 1:** Patient participants.

Pseudonym	Hospital setting	Gender
Simon	Emergency	Male
Stevens	Emergency	Male
Luca	Intensive Care	Male
Adrien	Intensive Care	Male

**TABLE 2:** Nurse participants.

Pseudonym	Practice setting	Gender
Sandra	Emergency	Female
Anna	Emergency	Female
Luc	Intensive Care	Male
Maryse	Intensive Care	Female
Assane	Emergency	Male
Nicolas	Emergency	Male
Laure	Emergency	Female
Jeanne	Intensive Care	Female

### 2.3 Data Analysis

Data were analysed according to the stages proposed in GT: open and axial coding, integration and theorization (or conceptualization). (a) Open coding: identification of codes (keywords, short phrases that synthesise participants' words) through interview transcripts. Annotations and codes were inserted in the margins of the interview transcripts. Subsequently, a codebook was developed and utilised to systematically analyse the other transcripts. (b) Axial coding: grouping the various codes into more general, abstract concepts. It is important at this stage to analyse emerging concepts according to their contexts (e.g., gender, sex, culture, or social and political contexts). At this stage, a conceptual map was created using XMind software to organise and analyse the emerging concepts. (c) Integration: the theoretical integration of concepts and categories, inviting a comparison of emerging results with similar empirical literature and relevant theoretical frameworks. (d) Theorising (or conceptualising) the phenomenon under study: theorising is the development of an emerging theory from the central categories.

### 2.4 Qualitative Rigour

The scientific criteria proposed by Corbin and Strauss (2014) were followed in this research, including validity, credibility, adequacy of the research process, and empirical grounding. To ensure the quality of the study, we remained attentive to theoretical concepts and philosophical influences, without imposing these frameworks on the collected data. Additionally, memos were written to document all analytical decisions. Emerging concepts were cross‐checked against participants' verbatim as well as empirical and theoretical literature.

### 2.5 Ethical Considerations

Ethical approvals for this study were obtained from the CIUSSS de l'Est‐de‐l'Île de Montréal's committee on research ethics and the Université de Montréal's Comité d'éthique de la recherche en sciences et santé (CERSES). Participants were asked to read and sign a consent form presenting information about the study (e.g., objectives, role of the researchers) prior to the interview. They were informed that they could withdraw from the study at any time, without being questioned by the research team. To ensure confidentiality, participants' names were changed to randomly generated pseudonyms.

## 3 Theoretical Perspectives

Within the grounded theory approach, it is possible to engage with theoretical frameworks, provided they are not imposed upon the data but rather used to inform interpretation in a flexible manner (Corbin and Strauss 2014). In this context, the analysis of empirical data draws on the work of the philosopher Gilles Deleuze (1992). In the 1990s, Deleuze followed up on Michel Foucault's writings on disciplinary systems (Foucault 1995), including psychiatry and justice. Deleuze considered these systems, or *dispositifs*, as characteristic of disciplinary societies, in which so‐called ‘at‐risk’ individuals are treated in secure settings such as psychiatric hospitals and prisons. Deleuze argued that disciplinary societies are in crisis; over the past few decades we have been witnessing a shift towards ‘societies of control’, where the population is under constant surveillance, not only in detention, but broadly, throughout society, via various control or ‘security apparatus’ (Razac 2008). According to this philosophical perspective, the mental health review board can be conceptualised as such a social control mechanism: people live in relative freedom, in society, but remain under ongoing surveillance by psychiatric and justice systems.

## 4 Results

Out of the qualitative analysis, we identified three central categories: (1) Involuntary (re)admission process; (2) Complexities of the care partnership; and (3) Ethical issues and prejudice. Below, we present these categories, accompanied by excerpts from participant interviews.

### 4.1 Category 1—Involuntary (Re)Admission Process

Interview participants shared their perceptions and experiences of the psychiatric admission process under a review board mandate. Some participants were incarcerated for a certain period (a few days to a few weeks) before being transferred from prison to the psychiatric hospital for assessment or admission.

I remember, they came to my house, in fact they rang the bell, but I knew it was them, I was waiting for them. At that moment, I knew I was going to prison. It was something I had expected and accepted in my head… Then, well, they grabbed me, pulled me out into the corridor, pinned me against the wall and handcuffed me. I should have told them that I didn't want to resist, I should have told them verbally that I wanted to go before the judge and that I was ready to go inside. But they roughed me up a bit, although it wasn't necessary. I know I didn't actually tell them that I was ready to surrender without resistance. After my violent attack on my neighbour, I can see how it was a tense situation and they might have been a bit afraid of how I might react. (Simon, user)

Such contact with the police following the commission of a crime while in a state of psychosis lead to a forensic assessment process in court, marking the beginning of these individuals' path through the psychiatry‐justice interface.

In the next excerpt, a nurse participant emphasises the importance of hospital care for people who have committed a crime for which they have been found not criminally responsible. This care is provided in a more appropriate clinical and therapeutic context than prison, where mental health care is more limited and the anxiety‐inducing environment is marked by violence and criminality.

Having a mental health disorder can lead you to commit certain crimes. There are some who have committed crimes in their past, but they're lucky enough to be treated in a hospital, versus being in a prison, doing your time … where you're not treated. So that at least, I dare to hope, is a positive thing for these people. (Jeanne, nurse)

#### 4.1.1 Police Involvement

As seen in the previous quotes, participants under a MHRB order reported having altercations with police officers during crisis situations. The intervention of law enforcement—in situations where the disturbed mental state of these individuals increases the risk of aggression they present to themselves, their entourage, or others—is, however, traumatic and can trigger previous experiences that are difficult to recall. Participants also expressed frustration with the involvement of the police in their psychiatric admission process.

It's always the same thing, always the same order … for example, the last time I came here it was for… I wrote on Twitter and, I wrote on Twitter and the police arrived at my house, and I ended up here. (Luca, user)

I stopped taking my medication, and then I became a bit fucked up, and that scared her [my wife]. And then I was arrested by the police the same evening, because we'd had an argument, and they took me to hospital. (Adrien, user)

These episodes of crisis and risk of violence, where symptoms of psychosis and mental health deterioration were present, meant that people were cared for in a secure hospital unit, for durations ranging from several weeks to several months for some participants. Following treatment, some participants were able to return to the community, on conditional release and under the supervision of outpatient teams, while others remained in detention.

#### 4.1.2 Mental Health Review Board Conditions

Individuals who are monitored in the community must follow the procedures imposed by the MHRB: most of these conditions relate to not using drugs, not disturbing the peace, attending follow‐up appointments with treatment teams, and following the prescribed care and treatment plan. It is interesting to note that the issue of medication intake and compliance is central in the discourse of users and nurses alike, even though the MHRB has no jurisdiction over treatment, and can therefore not impose a condition relating to it. For this nurse participant, the therapeutic context provided by both the inpatient hospital setting and intensive follow‐up in the community establishes a clinical framework that helps to ensure the mental and psychosocial health.

We work closely with them while they're in the unit and then gradually return them to the community. Except that when we return them, yes, according to the conditions, it has to be in a place where the doctor knows where they live. In other words, it really depends on each individual's circumstances. So, yes, they can be decompensated, or they can… how shall I put it … break a law: for example, if it is forbidden to approach a member of the family, or a spouse or … the place of residence, and he/she shows up. Yes, we must apply the law here too. We'll have to bring him in. (Assane, nurse)

Community follow‐up and the obligation to comply with the conditions may be complicated by a precarious socio‐economic environment, the living environment and issues of substance use and criminality.

I had external follow‐up but … basically, I'd managed to get a conditional release, with my treatment team. But I still had a hard time showing up for my appointments. I was too unstable. I don't like it too much, I cooperate, but I keep my distance, because my environment is less … outside, there's a lot of crime… Because I sell drugs, I value myself more in that. (Adrien, user)

#### 4.1.3 Delegations of Authority

Another aspect addressed by the nurses concerned delegations of authority. They explained that it is possible to readmit people who are the subject of such a court delegation. Depending on the person's legal status, treatment teams can apply the delegation of authority to readmit the person without going to court if they present a significant risk to public safety, and the risk cannot be managed in the community.

We have a book at the reception desk to keep track of TAQ patients… Do they have to be kept in hospital, or do they have the right to go out into the community and come back to the hospital, or do they have the right to go and live in an environment that is known to the team… (Assane, nurse)

In this context, it is important for nurses to be aware of the legal status of their patients. Knowledge of the legal notions that intersect with mental health care is essential to the practice of the nurses we interviewed. It is important for them to understand the different elements of judicial mechanisms (e.g., delegations of authority, the different courts). Some nurses spoke of the complexity of knowing the details and subtleties of legal procedures. For users, these court‐related specifics are even harder to grasp, because of the legal jargon and terminology.

So, from my point of view, of the information that's useful to me, I think I've got the main thing. After that, I think it could take years to fully understand all the legal issues involved. I think I'll still need time to … for sure there are specific things I haven't yet fully mastered. (Sandra, nurse)

### 4.2 Category 2—Complexities of Care Partnerships

Providing care to people involved with the justice system can be more difficult in some cases because of the involuntary nature of hospitalisation and the imposed therapeutic procedures.

In fact, in terms of the care we can provide, from the moment we force someone to be here, for me, we're pretty limited, i.e., I can't treat, or at least I can't provide… we can't be in a true partnership of care if I have someone who doesn't consent. (Sandra, nurse)

The ability to establish contact with people under a court order may be different, but it is still possible to forge bonds of trust and develop an alliance over time. Clinical experience and peer‐to‐peer learning foster relational skills.

I'd say I still manage to make contact. Of course, there are those who feel that the relationship doesn't go any further, but there are those who feel it's just right: ‘Hi, how are you today? What can I do for you?’ ‘Well, you can't do anything’. ‘Do you know why you're here?’ I explain the reasons. I try to assess their symptoms, if they allow me this access. And then the orders for treatment and accommodation, well, often it's injections, so it's a matter of seeing whether they've taken their medication, how they're doing, and then talking about discharge with the team afterwards. With the TAQ, it's the same thing. (Anna, nurse)

Here we see the importance of building bonds of trust, despite the relational barriers that may be encountered. Certainly, some users may mistrust or even be frustrated with the psychiatric milieu.

#### 4.2.1 Denial of Illness

The complexity of establishing a care partnership may also be due to the non‐recognition of symptoms among hospitalised individuals. Lack of insight and loss of contact with reality can lead to denial of the illness and the need for psychiatric care.

It's part of our daily reality. Some of our patients are under MHRB orders. So what this means for us, well, it's clear that when a patient knows there is an application for custody, or when he goes to see the judge and then comes back and it's decided, it's rarely positive. You have to deal with users' reactions, and sometimes incomprehension, because: ‘I'm not ill, I don't need to be here’… And that's a big part of our job. (Jeanne, nurse)

In the same vein, it has been pointed out that some of the people admitted manage to minimise the symptoms, and do not recognise the diagnosis or the offence committed. It was suggested that the aim here could also be to remove the court order and the annual or biannual review in place.

I would say that what I often see with TAQ patients is a lot of denial. When we talk to them about: ‘You're under a TAQ order’. You don't have to appear in court, but we keep you in detention under delegated authority, because something happened and everything… But, often, I would say for more than 50 to 75% it's: ‘No, it's not true’.… They're often in denial, or they explain it away or minimize the event that brought them to the TAQ… It's rare for them to admit: ‘yes, it's true that I did that’. That's very very rare. (Luc, nurse)

Issues related to drug use were also raised in the interviews. In addition to the court‐ordered prohibition on drug use, it turns out that for these people, the substances they consume are likely to further deteriorate their mental state, putting them at risk of readmission.

But when I take drugs on top of that, it doesn't help, I get all fucked up… And I'm bipolar, I take medication for it. But I went into a manic episode, I hallucinated. I was listening to music, I thought the music was speaking to me. I started to distrust everyone, I started to look in the street, to look for a gun, to do things … to want to protect myself. (Adrien, user)

With regard to denial of the illness, one participant spoke of the journey he had made towards recognition of his state of psychosis, and the need for hospitalisation and care.

Well, I knew I was in trouble. But, I still didn't understand that I was in psychosis, I thought I was right, I thought people were plotting against me, really. I defended myself to the judge right to the end. Then, well, finally, I was transferred to a psychiatric facility for mental illness. Then, at some point, I began to realize that I was imagining things. Like I was imagining things that weren't real. (Simon, user)

#### 4.2.2 Compliance With Treatment and Care Plan

In addition to denial of illness and the diagnosis, lack of compliance with pharmacological treatment emerged from the interviews as an issue widely encountered by psychiatric nurses.

I'd say around a third of users … around a third of users are there on a voluntary basis. Two thirds are not voluntary patients, either because they come under a TAQ order, because of what we call delegations of authority, or because they come under a treatment order. So, they had stopped their treatment… And it's even harder, the alliance, to work with these individuals. For them to collaborate with us. To accept treatment, and work together to shorten hospital stays. (Luc, nurse)

Some of the users spoke of the difficulties involved in taking their medication, which for many is done under court authorization (e.g., community treatment order). This use of medication (often long‐acting injectables) prompts reflection on the benefits to their health, but also on the side effects and loss of autonomy that this may entail.

But when I take my medication I'm normal, I'm a different person. But when I don't take them… I don't know … but it's annoying, it's annoying to be confronted with medication like that and feel a bit … like a puppet, like, inside. To have to get an injection every month, and so on and so on. It seems like it's always something else, and you start shaking because of the medication, and they have to add another medication for the shaking, and it never ends. (Adrien, user)

The data analysed also indicate that in order to maintain a bond with involuntarily (re)admitted people, it's important to remain empathetic, to understand the other person's lived experience and to focus on a non‐judgmental, humanistic approach.

For me, it's all about empathy. Because the idea of receiving a patient is one thing, but it's important to me that… I establish a bond of trust with patients. (Nicolas, nurse)

I always try to find the human element behind every illness. Then I try to connect with the human being behind it. Yes, you're here for a mental health disorder, but that doesn't define you. The more I focus on their hopes, their life goals. (Jeanne, nurse)

### 4.3 Category 3—Ethical Issues and Prejudice

The final category concerns ethical issues, in addition to the harm experienced by users. For nurses, the ethical issues and dilemmas mainly concern the care of persons admitted or treated involuntarily, and the difficulty of acting both as a caregiver and as an agent of social control. The complexity of providing care for NCR patients may be at the root of reflections and certain questions regarding non‐criminal responsibility. For some, these reflections concern the merits of the process and the increase in verdicts of NCR that they observe in their practice.

There are some patients who play the card of, well you know: ‘I'm sick when it suits me, and I'm not sick when it doesn't suit me’. That's really frustrating, because we're seeing more and more… I think that's the negative side of this system. But otherwise, it's good to have something to protect them, with a more understanding context of what illness is. (Jeanne, nurse)

Agreeing to treatment, attending appointments when under care in the community and complying with court obligations are factors that can be a source of dilemmas for users. As already mentioned, they face the requirement of taking medication regardless of the side effects they may experience. In the next excerpt, a participant comes to regret having been declared not criminally responsible for the offence committed, because of the constraints and obligations this entails for him. With hindsight, he considers that a prison sentence—with a fixed and known duration—might have been a lesser evil than the current situation.

They tell me I'm not criminally responsible. I don't care if I'm criminally responsible. I want to be criminally responsible, so I don't have to deal with the TAQ, because I'm mentally OK. I don't need injections to put in my body any more. (Stevens, user)

In addition to the ethical issues confronting them, participants spoke of the harm suffered by those under the supervision of the mental health courts. They stated that repeated hospitalizations and imposed conditions make finding a job more complex, in addition to putting individuals at risk of social isolation when they live in the community.

Users who are under both systems [mental health and justice] are often the most vulnerable … that's what I've noticed, they're maybe more isolated and also quickly slip into drug use. (Jeanne, nurse)

Before it [the review board] was new, and it did something to me. Because I thought I was crazy, I thought … and I lost friends in there too, like from being hospitalized. (Adrien, user)

Psychosocial difficulties experienced (e.g., exclusion and family breakdown) were reported as consequences for some. The lack of consideration for the experience of people under MHRB mandate was also mentioned, as was the loss of autonomy and control over one's own life.

I don't know, it takes away self‐esteem. It broke me a little. (Adrien, user)

The TAQ is always … what the doctors say, it's always what the police say, it's always what other people say. I lose my rights all the time. Totally… I've lost any right to my opinion, decision‐making power. (Lucas, user)

## 5 Discussion

Our results show that the reality of individuals under a MHRB mandate is complex and influenced by various coercive measures and disciplinary mechanisms. These include altercations with the police, time spent in detention, and the obligation to comply with rules that govern every part of their lives. Our analysis reveals that people undergoing review board follow‐up have the impression of being caught in a vicious circle, with coercive oversight extending over the long term. Interviews with psychiatric nurses also raised the complexity of providing care to patients in situations of involuntary admission. The results of our study indicate that the admission or readmission process for NCR individuals occurs in tandem with community‐based care: in the event of non‐compliance with the procedures in place, a mechanism allows for them—in certain contexts—to be readmitted to a secure setting, usually with a police escort.

In terms of ethical issues, other studies have also focused on the experience of care providers. Merkt et al. (2021) explored the conflict of allegiance experienced by clinicians in the context of court‐mandated psychiatric readmission. This conflict arises, on the one hand, from the need to abide by the law and the importance of protecting the public, and on the other, the professional duty to advocate for the interests and rights of users. The literature also highlights a certain ambivalence among clinicians. On the one hand, the legal framework provided by monitoring and care procedures is seen as positive and supportive (Riordan et al. 2005), improving treatment compliance and enabling early identification of psychosis relapse (Reynolds 2023). Other studies suggest, however, that legal coercion is a significant barrier to building trust between clinicians and users (Haines et al. 2024), as confirmed by participants in our study. Clearly, the care provided to individuals under judicial mandate is shaped by unequal power dynamics and generates numerous dilemmas for all actors involved.

Qualitative studies have also focused on the experience of individuals undergoing involuntary readmission. In England, a grounded theory (Chiringa et al. 2014) was carried out with people (*n* = 6) under a review board order who had been readmitted after breaching conditions. The findings showed that people often feel at the mercy of the psychiatric and justice systems, which they perceive as unfair and overly controlling. Another British study (Rye et al. 2019) reported on the feeling of powerlessness and persecution experienced within the forensic system, which exacerbates the already limited involvement of users in their care and treatment. Similar to our findings, Rye et al. (2019) reported that forensic psychiatric care considerably disrupts users' personal and social lives (e.g., family disruption, job loss). The lack of community support from mental health services was also raised as an obstacle to their community reintegration.

Another finding of our study is the complexity of building trust‐based relationships with those admitted, some of whom are reluctant to seek mental health care. Intensive monitoring and judicial supervision can lead to mistrust (Riordan et al. 2005), frustration, and resentment towards health care professionals (Chiringa et al. 2014). Participants also reported feeling dependent and under tutelage (Whittaker et al. 2023). In an ethnographic study, Sallée et al. (2022) state that justice‐involved individuals have little to say in the decision‐making process, which severely restricts their freedom and autonomy.

At a theoretical level, authors have explored how social control modalities—such as monitoring by the MHRB—affect all facets of the lives of people living with mental health problems (Dixon 2015; Sallée et al. 2022). We analysed how medico‐legal follow‐up involves care activities and disciplinary power techniques. These include incarceration, regular court appearances, the imposition of strict conditions, and limitations on unescorted absences or release. These mechanisms, simultaneously coercive and therapeutic, enable constant surveillance and control of individuals in all aspects of their lives, both inside and outside the hospital environment.

The MHRB operates in a way similar to Deleuze's concept of ‘societies of control’ (Deleuze 1992). A person monitored by the review board, even while living in the community, remains under the constant supervision of the treatment team. If the person poses a significant risk to public safety that cannot be managed in the community, he or she may be readmitted to secure custody, where he or she will once again be subject to disciplinary mechanisms (surveillance and involuntary administration of medication in most cases). The threat hanging over these individuals in the event of an eventual failure to comply with the conditions laid down by the courts (e.g., non‐compliance with treatment and follow‐up appointments, drug and alcohol use) can lead to involuntary psychiatric readmission, often with police intervention. We also observed that medication compliance is a crucial aspect of care for these individuals, and often an essential condition for assessments conducted by the nurses we interviewed. The issue of psychopharmacology is similarly central in other studies (Almeida et al. 2016; Chiringa et al. 2014; Jewell et al. 2018), particularly in decisions handed down by review boards (Bernheim et al. 2023; Sallée et al. 2022).

Justice‐involved individuals must accept a more ‘human’ form of supervision within society, or risk being involuntarily readmitted. They are encouraged to regulate their own conduct and to participate actively in their treatment plan. They must accept conditional follow‐up as a necessary evil, preferable to the sword of Damocles represented by psychiatric admission. In this way, they may be able to live in the community—or at least obtain authorised release—while constantly knowing they can be readmitted if they fail to comply. In societies of control, the transition between the inside (*striated spaces*) and the outside (*smooth spaces*) (Deleuze and Guattari 1987) takes place transversally, with a continuous back‐and‐forth movement between different surveillance spaces. According to Foucault (1995), the medico‐legal terms used to describe these individuals (e.g., high‐risk accused, NCR patients), along with the control mechanisms in place (conditional release, constant surveillance, delegation of authority), reshape their identity and contribute to their subjection to the ‘regimes of truth’ inherent to psychiatric and legal systems.

In interviews, we were informed that MHRB hearings occur annually and can be repeated over several years. Those subject to this legal process know when the sentence begins, but its duration remains indeterminate and fluctuates according to the risk the person presents to the public. One of the defining attributes of societies of control is diffuse temporality: ‘in the societies of control one is never finished with anything’ (Deleuze 1992, 5). Some participants expressed a preference for being declared criminally responsible and given a short prison sentence, rather than subjected to a more empathetic and therapeutic form of continuous surveillance with an unknown end date, one that scrutinises and regulates their every move. In conclusion, by including in this research individuals under a MHRB order, we have been able to amplify the voices of a vulnerable population that is often underrepresented in studies. To inform health care decision‐makers and nurses about the practices affecting these vulnerable groups, it is crucial to consider the lived experience and perceptions of these populations, as well as those of the clinicians who work with them.

## 6 Limitations

This study was conducted with individuals under the jurisdiction of the Mental Health Review Board, specifically during periods of involuntary hospitalisation in a psychiatric institution. We therefore did not include persons receiving community‐based care, which could have broadened the analysis and understanding of the phenomenon. In addition, the sample of users included only four participants, all of whom were male. Future research should aim to recruit a more gender‐diverse sample to better explore the sex and gender dimensions associated with judicial trajectories following a NRC verdict. Lastly, transcripts were not returned to participants for external validation, which could have contributed to the analysis process and the criteria for rigour in qualitative research.

## 7 Relevance for Clinical Practice

Supervision by mental health review boards adds complexity to the treatment and nursing care provided to people with mental health conditions. The results of this qualitative study may contribute to the development of clinical interventions that are specifically tailored to the needs of individuals admitted to and monitored by the justice system. It is important for mental health nurses to better understand the impact of justice system involvement on the patients they care for. This understanding could lead to more appropriate and effective support.

## Author Contributions

Etienne Paradis‐Gagné: contributed to the realisation and coordination of the study and the writing of the manuscript. Marie‐Ève Roy‐Ducharme: participated in data analysis, manuscript writing, and revision. Manuel Argod: contributed to the writing and revision of the manuscript. Emmanuelle Bernheim: contributed to the project funding application, project completion, data collection, and manuscript revision. Dave Holmes: contributed to the project funding application, project completion, data collection, and manuscript revision.

## Funding

This research was supported by the Canadian Institutes of Health Research (CIHR)–grant no. 178312.

## Ethics Statement

Ethical approvals for this study were obtained from the CIUSSS de l'Est‐de‐l'Île de Montréal's committee on research ethics and the Université de Montréal's Comité d'éthique de la recherche en sciences et santé (CERSES).

## Conflicts of Interest

The authors declare no conflicts of interest.

## Data Availability

The data that support the findings of this study are available on request from the corresponding author. The data are not publicly available due to privacy or ethical restrictions.
